# Highly sensitive detection of estradiol by a SERS sensor based on TiO_2_ covered with gold nanoparticles

**DOI:** 10.3762/bjnano.11.87

**Published:** 2020-07-14

**Authors:** Andrea Brognara, Ili F Mohamad Ali Nasri, Beatrice R Bricchi, Andrea Li Bassi, Caroline Gauchotte-Lindsay, Matteo Ghidelli, Nathalie Lidgi-Guigui

**Affiliations:** 1 Dipartimento di Energia, Laboratorio Materiali Micro e Nanostrutturati, Politecnico di Milano, via Ponzio 34/3, I-20133 Milano, Italy; 2 Department of Structure and Nano/-Micromechanics of Materials, Max-Planck-Institut für Eisenforschung GmbH, Max-Planck-Straße 1, 40237 Düsseldorf, Germany; 3 James Watt School of Engineering, Rankine Building, Oakfield Avenue, G12 8LT, University of Glasgow, Glasgow, United Kingdom; 4 now at Laboratoire des Sciences des Procédés et des Matériaux (LSPM), CNRS, Université Sorbonne Paris Nord, 93430, Villetaneuse, France; 5 CSPBAT, UMR 7244, Université Sorbonne Paris Nord, 93430 Villetaneuse, France

**Keywords:** aptamer, Au nanoparticles, 17-β estradiol (E2), plasmonics, sensor, surface-enhanced Raman scattering (SERS), TiO_2_ nanostructures

## Abstract

We propose the use of gold nanoparticles grown on the surface of nanoporous TiO_2_ films as surface-enhanced Raman scattering (SERS) sensors for the detection of 17β-estradiol. Gold deposition on top of a TiO_2_ surface leads to the formation of nanoparticles the plasmonic properties of which fulfil the requirements of a SERS sensor. The morphological and optical properties of the surface were investigated. Specifically, we demonstrate that the TiO_2_ background pressure during pulsed laser deposition and the annealing conditions offer control over the formation of Au nanoparticles with different sizes, shapes and distributions, yielding a versatile sensor. We have exploited the surface for the detection of 17β-estradiol, an emerging contaminant in environmental waters. We have found a limit of detection of 1 nM with a sensitivity allowing for a dynamic range of five orders of magnitude (up to 100 µM).

## Introduction

Surface-enhanced Raman scattering (SERS) as a sensing tool requires the optimization of a surface and its functionalization. The surface should provide a good enhancement over a large range of wavelengths, to detect molecules with various fingerprints, while it should also be easy to fabricate at reduced cost. In addition, the surface functionalization needs to guarantee the selection, detection and quantification of a target molecule, e.g., a biomarker [1–3] or a pollutant [4–5] dissolved in complex media such as blood, plasma, urine, or river and sea water.

SERS is mainly based on an electromagnetic effect that originates from the excitation of plasmon resonances, in particular of localized surface plasmons (LSPs) in metallic nanoparticles (NPs). Other effects may contribute to the enhancement such as the formation of hot spots or lightning rod effects [6–8]. Many surfaces were proposed for SERS including rough metallic surfaces [9–10], colloidal solutions [11], and structures with controlled size, distance and shape obtained via lithography techniques [2,5,12]. However, these techniques can be time-consuming and expensive. Recently, the use of composite systems of dielectrics (TiO_2_, ZnO) and metallic NPs has gathered increasing attention regarding SERS applications, because the plasmonic enhancement provided by metallic NPs can be combined with the optical properties of the semiconductor such as light trapping, scattering, and antireflection abilities [13–15]. These composite microstructures have also shown to maximize the path of the Raman excitation laser beam within the substrate, leading to signals with higher intensity.

Samransuksamer et al. [16] used TiO_2_ nanorods decorated with Au NPs, deposited via precipitation by soaking in HAuCl_4_ solution, as composite SERS substrates for the detection of methylene blue. They reported a successful SERS enhancement, compared to bare Si substrates, with an enhancement factor of ca. 10^6^ and a lower detection limit of 100 nM. Li et al. [15] studied Au NP-coated TiO_2_ nanotube arrays as SERS substrate for the detection of rhodamine 6G and other organic molecules. They obtained stable and reproducible results with a detection limit down to 10 µM, while also showing high recyclability through cleaning via UV irradiation. However, a main drawback of these methodologies is the use of aggressive solvents, which can induce damage especially in delicate applications involving polymeric substrates. Also, the control over the size and shape of AuNPs and, thus, over their plasmonic behavior is often limited.

Here, we propose the use of a nanostructured hierarchically organized TiO_2_ film as a template for the growth of Au NPs (in the following the samples will be referred as TiO_2_/Au). Both TiO_2_ film and Au NPs were synthetized by vapor phase deposition techniques (involving pulsed laser deposition and thermal evaporation) avoiding the use of solvents, while accurately tuning the morphology and the plasmonic properties. Specifically, TiO_2_ films with different porosities have been deposited, with different Au NP sizes and coverages. Then, the growth parameters of TiO_2_ and of the AuNPs were selected in order to obtain the maximum SERS enhancement. In a second step, the Au NPs were functionalized with aptamers (a biorecognition element), specific to the natural estrogen 17β-estradiol (E2) [17] for SERS detection. The all-solid configuration of the TiO_2_/Au surface makes it a good candidate for in situ detection.

Surface functionalization with aptamers is gathering interest because they possess many of the important qualities required for the functionalization of SERS sensors [18–21]. Aptamers are single-stranded DNA molecules that are specifically selected to bind to a target molecule. They are relatively cheap and their chemistry is easy to tune so that they can attach to a metallic surface. Also, they can be selected to be short enough to guarantee that the targeted molecule is in the enhancement volume of the plasmonic nanoparticle (the effect of SERS decreases exponentially with the distance from the surface and is negligible beyond 5 nm). Another interesting feature of aptamers is that their Raman fingerprint is easily recognizable, as DNA is an extensively studied molecule.

In this study, we focus on the detection of E2 with an aptamer-functionalized sensor. E2 is the main female hormone responsible for growth, reproduction, breast development, maturation, bone formation, and childbearing in mammals. It is the most potent estrogen [22]. Estrogens found in the environment originate from human and animal excretions and are released into surface waters from agricultural activities, non-treated waste or wastewater treatment effluents [23]. High concentrations of E2 have been found in surface and groundwater in urban areas, leading to rising concerns in the EU. Studies have revealed that in some fish species, exposure to E2 has led to the feminization of males [24–25]. Routine instrumental methods for the detection of E2 in environmental waters are well established [26]. They are very specific and very sensitive, however, they are also time-consuming and expensive [27]. SERS sensors are therefore investigated as an alternative as they present the potential for in situ near-real-time analysis.

In the following we will present the possibilities of TiO_2_ porous surfaces decorated with Au NPs regarding the use as SERS sensors. The tuning of TiO_2_ growth and Au deposition gives access to a variety of surfaces with specific optical properties. In a second part, we show that it is possible to detect as low as 1 nM of E2 using these surfaces.

## Experimental

### Growth of the TiO_2_/Au nanostructured surfaces

TiO_2_/Au substrates were synthetized using a two-step deposition. First, a nanostructured TiO_2_ film was synthetized by pulsed laser deposition (PLD). Then, a Au NP layer was deposited on top by thermal evaporation of Au followed by solid-state dewetting to induce the formation of NPs. A TiO_2_ (99.9%) target was ablated using a Nd:YAG laser (λ = 532 nm) with a pulse duration of 5–7 ns and 10 Hz repetition rate. The laser fluence on the target is 3.5 J·cm^−2^ and the pulse energy is 200 mJ. Film synthesis was carried out at room temperature in oxygen atmosphere, using both Si(100) and soda-lime glass substrates, which were mounted on a sample holder at a fixed target-to-substrate distance of 5 cm. Changing the background pressure within the deposition chamber allowed for a tuning of the film morphology (i.e., a higher pressure resulted in a higher film porosity) [28–29]. Samples were therefore deposited at a pressure of 8 or 12 Pa to obtain different film porosities.

A thin layer of Au (99.9%) was then evaporated using an Edwards E306A resistively heated thermal evaporator. The equivalent (i.e. nominal) thickness of the evaporated layers was monitored with a quartz microbalance sensor. Three different values of Au thickness, namely 3, 6, and 15 nm, were selected enabling the formation of different sizes of AuNPs through subsequent annealing. Selected samples underwent annealing at 500 °C for 2 h in air, in a Lenton muffle furnace with 4 °C/min heating ramp. The thermal treatment was carried out to induce both crystallization of the as-deposited amorphous TiO_2_ into the anatase phase (as discussed in [28–29]) and the formation of AuNPs exploiting dewetting of the Au films.

A field-emission scanning electron microscope (FEG-SEM, Zeiss Supra 40) was used to perform morphological characterizations of the films deposited on Si(100) substrates. The average size distribution of Au NPs was estimated through statistical analysis of top-view SEM images with the open source software ImageJ^®^. Since the shape of the Au NPs was not always perfectly circular, their area was measured with ImageJ^®^ in order to calculate the equivalent diameter, which was used to define the NP size.

### Chemicals and reagents

Mercaptobenzoic acid (MBA), 6-mercapto-1-hexanol (MCH), 17β-estradiol (E2), and ethanol were purchased from Sigma-Aldrich.

A 17β-estradiol binding aptamer was previously isolated by the SELEX process by Kim and co-workers [17]. It was purchased from Eurogenetec with the following 76-mer-long sequence SH-C6-5′-GCT-TCC-AGC-TTA-TTG-AAT-TAC-ACG-CAG-AGG-GTA-GCG-GCT-CTG-CGC-ATT-CAA-TTG-CTG-CGC-GCT-GAA-GCG-CGG-AAG-C-3′. The thiol group was added to the 5′ end of the aptamer for functionalization via a Au–S bond. The aptamer was diluted and aliquoted upon arrival and stored at −18 °C.

### Sample functionalization

MBA was used as a common Raman reporter to find out the best deposition parameters for Raman scattering enhancement. The thiol group is known to have a strong affinity to Au but none to TiO_2_. Moreover, the Raman spectrum of MBA is well known and the molecule demonstrates a large scattering cross section [30]. For the functionalization of TiO_2_/Au surfaces, MBA was diluted in ethanol at a concentration of 2.9 mM. The TiO_2_/Au samples were then soaked in the solution overnight. After that, they were thoroughly rinsed with ethanol before drying with nitrogen.

**Figure 1:**
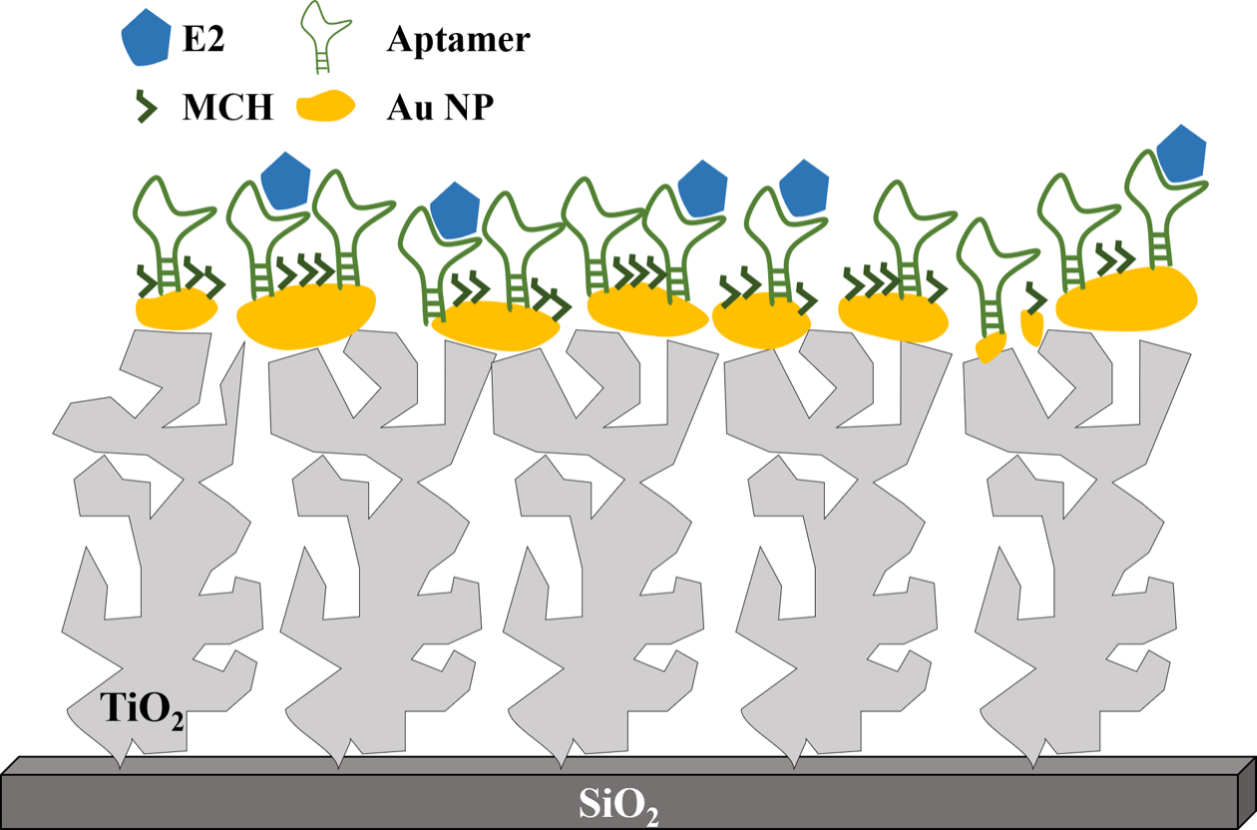
Schematic of the SERS sensor. The figure is not to scale for the sake of clarity.

For E2 detection, the TiO_2_/Au surfaces were first functionalized with the aptamer. For this a fresh 3 μM solution of aptamer was prepared in Tris-HCl buffer solution (20 mM Tris-HCl contained 1 M of HCl, 0.1 M of NaCl and 5 mM of MgCl_2_, pH 7.5). The samples were left for 2 h in this solution before rinsing with Tris-HCl buffer solution and Milli-Q water. In order to prevent unspecific interactions between E2 and the gold surface, the sample was then left for 2 h in a solution of the blocking agent MCH. MCH occupies gold sites that are not functionalized with the aptamer (Figure 1). This prevents the deposition of unwanted molecules from the sample, which could blur the signal. It also prevents the amine groups of DNA to form weak bonds with the gold and it helps the aptamer to have a homogeneous orientation on the surface [31].

The MCH solution was prepared in phosphate-buffered saline (PBS) solution mixed with 10 mM MgCl_2_. The concentration of MCH was constant at 14.6 μM. The samples were rinsed three times with PBS and reverse osmosis water (RO water) to remove any unbound or excess molecules after the deposition.

Samples with different concentrations of E2 were prepared in order to test the sensitivity of the sensor. E2 was first diluted in ethanol at saturation (36 mM). This solution was further diluted in RO water to obtained E2 solutions with concentrations of 1 nM, 10 nM, 100 nM, 1 µM, 10 µM, 100 µM, and 1 mM. Samples were left in the E2 solutions for 1 h before being rinsed with RO water and blown dry. Figure 1 gives a schematic of the final system.

### Optical and SERS measurements

Plasmon resonance was evaluated via optical spectroscopy. For this purpose, transmission spectra were acquired using a UV–vis–NIR spectrophotometer (PerkinElmer Lambda 1050) with a 150 mm diameter integrating sphere in the range of 250–2000 nm, illuminating the sample from the glass substrate side. All acquired spectra were normalized with respect to the contribution of the glass substrate.

The SERS spectra were recorded with a micro-Raman spectrophotometer (Jobin-Yvon Labram 300), using a 100× magnification objective (NA = 0.90) in back-scattering geometry, with a spectral resolution of 3 cm^−1^ and a spatial resolution of about 1 µm. The employed excitation wavelength was 633 nm, with a power of 1 mW for an acquisition time of 300 s. This trade-off between power and duration of the acquisition has been chosen after a series of tests in which the power was gradually decreased from 10 mW to 100 μW. The typical peak of silicon at 521 cm^−1^ was used as an internal reference to normalize the intensities of all the spectra. The spectra presented here are the average of four spectra taken at different locations of each sample.

## Results and Discussion

### Sample growth and structural characterization

#### Morphology

Nanostructured TiO_2_ films with hierarchical micrometer/nanometer-scale morphology and tuned porosity were deposited by pulsed laser deposition (PLD) as already reported in [28]. By increasing the background O_2_ pressure during deposition it is possible to deposit films that are more porous. Samples were therefore synthetized at background pressures of 8 or 12 Pa. Au layers were then evaporated on top of the TiO_2_ films. Three nominal thickness values of 3, 6, and 15 nm were chosen, in order to obtain NPs with different diameters (Table 1). After deposition of Au, samples underwent an annealing treatment in a furnace at 500 °C for 2 h, which leads to the crystallization of TiO_2_ to the anatase phase (as demonstrated by Raman spectra, not shown) and caused dewetting in the Au layer with the subsequent formation of NPs [32]. Evaporation and dewetting of Au on TiO_2_ layers with different porosity was carried out to exploit the effect of the surface morphology on the formation of Au NPs, yielding different size distributions and densities.

**Table 1:** Average equivalent diameter with standard deviation of Au NPs on TiO_2_ films after annealing.

	8 Pa			12 Pa		
	Au 3 nm	Au 6 nm	Au 15 nm	Au 3 nm	Au 6 nm	Au 15 nm

average diameter [nm]	12	23	115	13	19	64
standard deviation [nm]	4	12	88	6	13	42
Au surface coverage [%]	9.5	21.6	35.1	9.4	22.2	35.7

**Figure 2:**
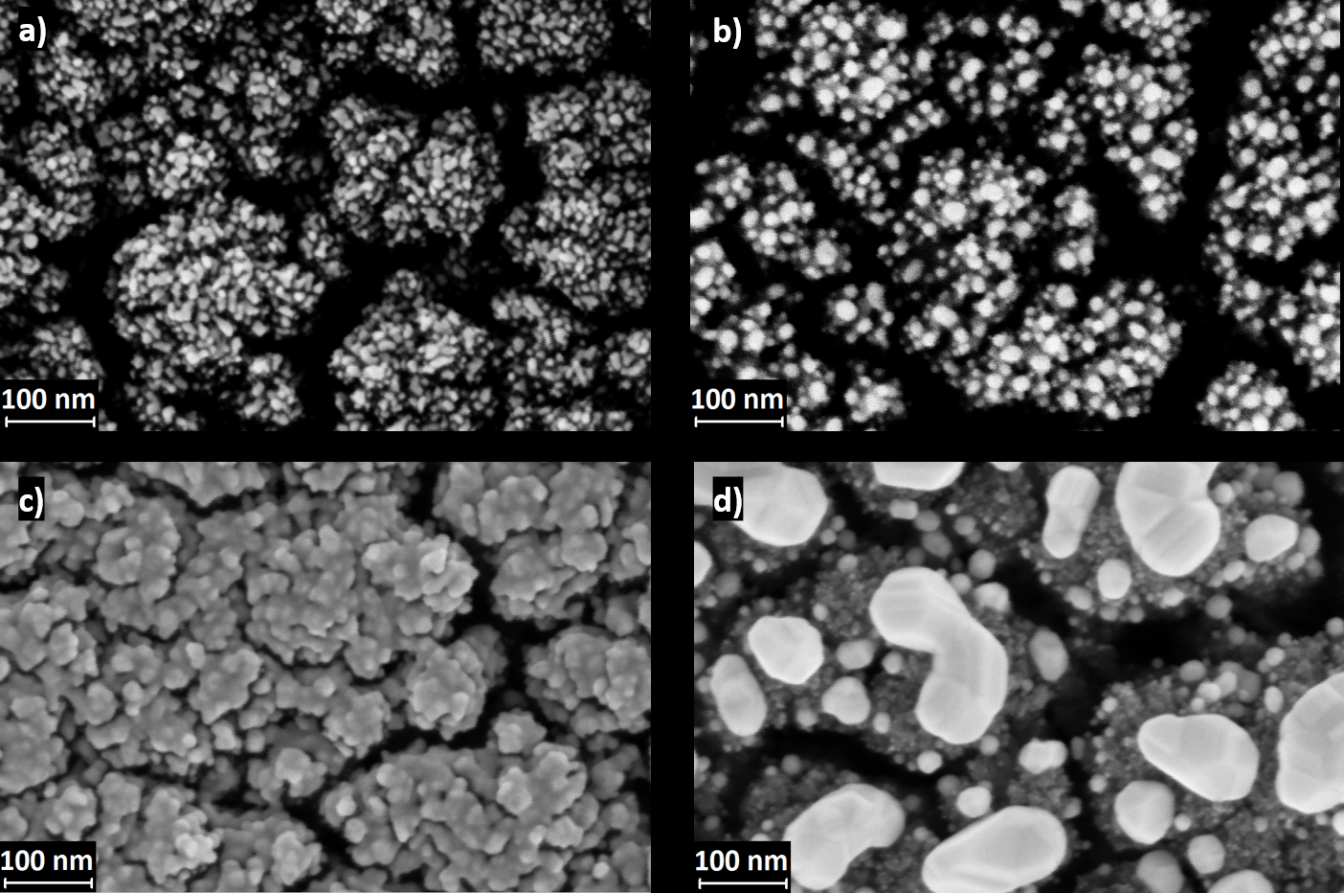
SEM top-view images showing 12 Pa TiO_2_ samples with 3 nm of evaporated Au before (a) and after (b) annealing. Panels (c) and (d) show 12 Pa TiO_2_ samples with 15 nm of evaporated Au, respectively, before (Au continuous layer) and after annealing (completely formed NPs).

Figure 2 shows the two boundary cases of 3 and 15 nm of evaporated Au on a TiO_2_ film deposited at 12 Pa. The observations were similar for the 8 Pa films. The effects of heat treatment are clearly visible. In the case of 15 nm of Au (Figure 2c,d), a continuous layer is formed on the underlying TiO_2_ surface, which upon thermal treatment forms isolated, large Au nanoislands. Evaporation of 3 nm of Au resulted instead in a non-continuous nanostructured layer, for which thermal treatment led to the growth of well separated AuNPs (Figure 2a,b).

**Figure 3:**
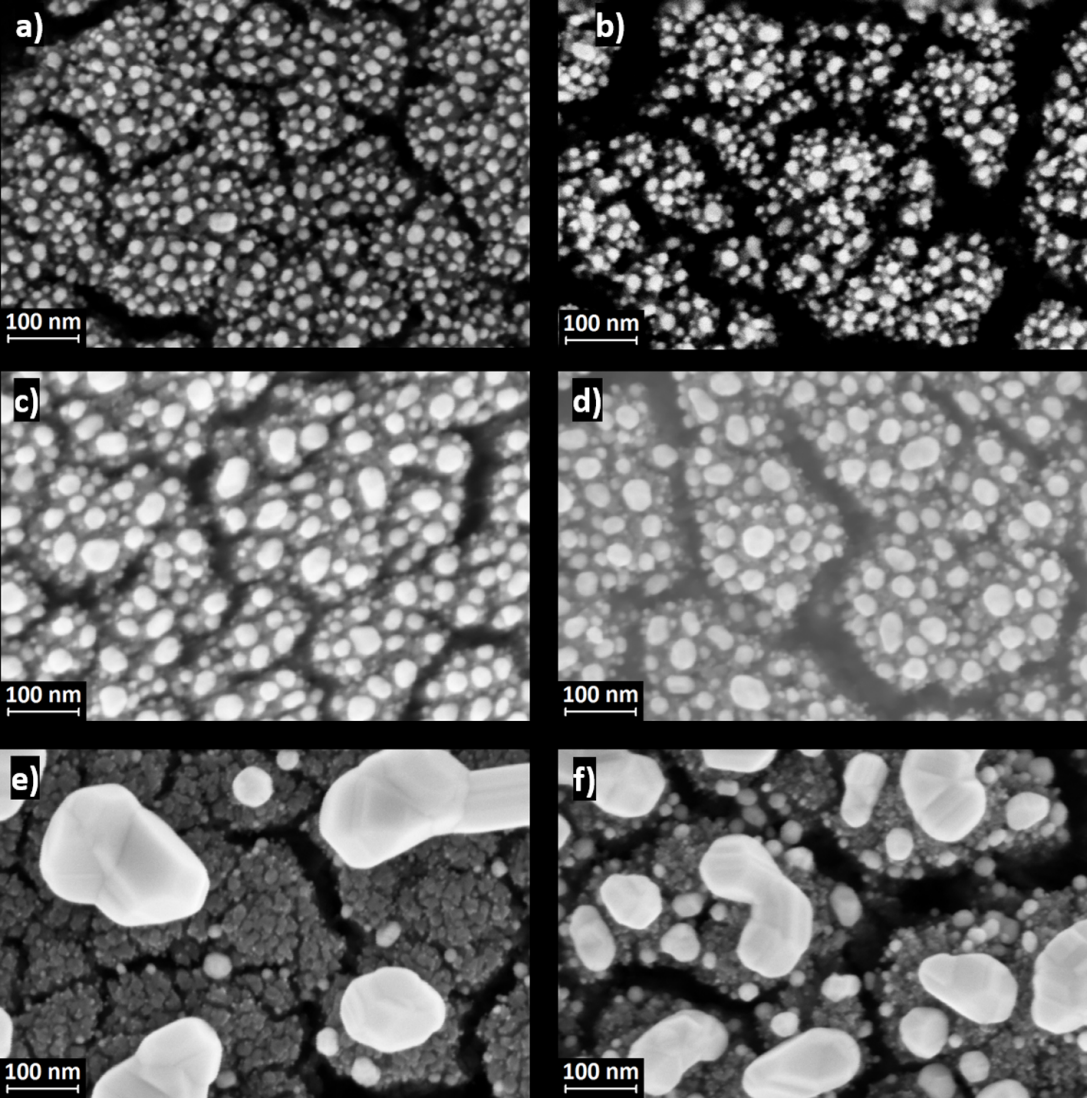
SEM top-view images of TiO_2_ 8 Pa with 3 nm (a), 6 nm (c), and 15 nm (e) of Au, and of TiO_2_ 12 Pa with 3 nm (b), 6 nm (d), and 15 nm (f) of Au. All micrographs were acquired after annealing.

Statistical analyses of the top-view SEM images after annealing (some of which are reported in Figure 3) proved that by increasing the amount of evaporated Au, it was possible to increase the NP size. The average equivalent NP diameters varied from 12 up to 115 nm (Table 1). Moreover, the morphology of the TiO_2_ film also played a role in determining the final Au NP diameter, with smaller equivalent diameters obtained in the case of more porous films deposited at 12 Pa. Finally, the Au coverage increased with the amount of Au deposited on the TiO_2_ surface up to almost 30%, while it was almost independent of the TiO_2_ porosity.

#### Optical properties

**Figure 4:**
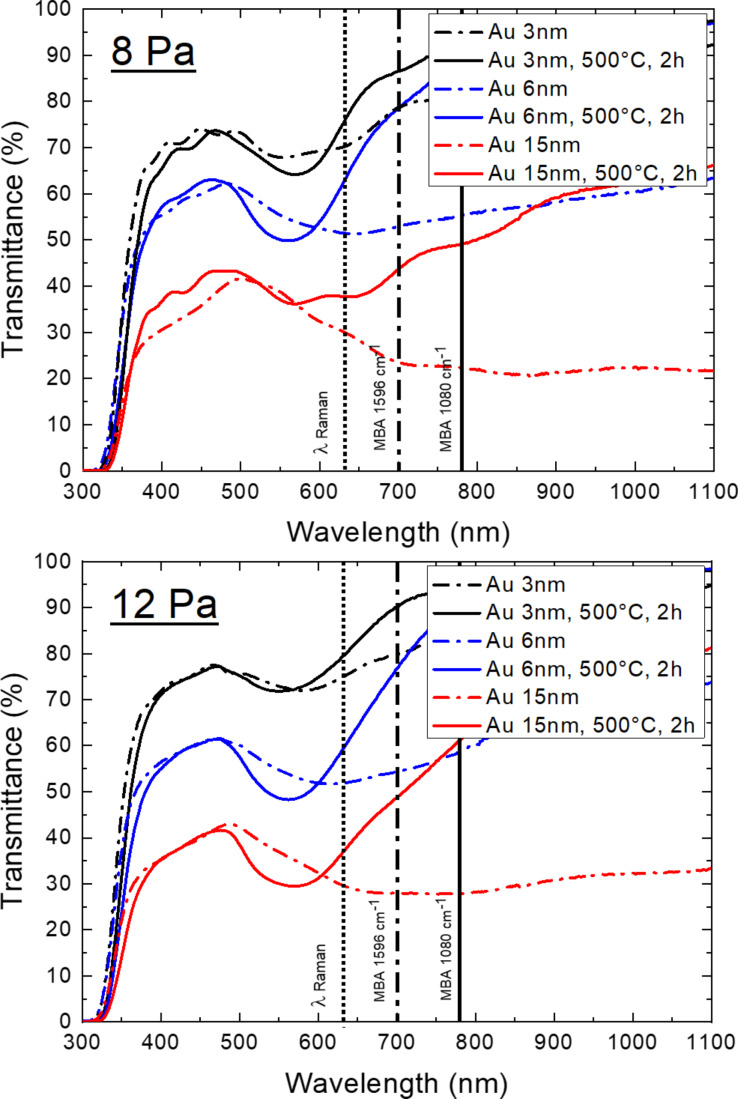
Optical transmission spectra of TiO_2_/Au 8 Pa (a) and 12 Pa (b) samples before and after annealing.

Figure 4 reports the optical transmittance of the TiO_2_/Au samples. Our previous studies involving TiO_2_/Au systems have shown that the reflectance has low values of about 5% and can thus be considered negligible [28]. A general decrease of the optical transmittance was observed for large quantities of Au and the transmittance was influenced by the growth conditions and the thermal treatment. For example, in the case of 6 nm of Au the wavelength at which maximum absorption occurs changed after annealing from about 620 to 560 nm (Table 2). These trends were outlined also in works by Dorono-Mor et al. [33] and Karakouz and co-workers [34]. More specifically, the as-deposited TiO_2_/Au samples displayed a very broad absorption, especially for greater amounts of evaporated Au, when an almost continuous Au layer was formed. However, after annealing the absorption peak became sharper and blue-shifted, which can be attributed to the formation of Au NPs. In the annealed samples the LSP resonance (LSPR) red-shifted as a function of the Au NP size, i.e., the wavelength for maximum absorption varied from 549 nm (for the 12 Pa sample with 3 nm of Au) up to a maximum value of 575 nm (for the 8 Pa sample with 15 nm of Au) [35]. Moreover, the full width at half maximum (FWHM) increased as a function of the AuNP size, due to the fact that NPs exhibited a broader dispersion, as reported also by Gaspar and co-workers [36]. In other words, the optical properties and the LSPR changed due to the morphology change of the deposited Au, from an almost continuous layer to various shape/aspect ratios, size distributions, and average distances between the Au NPs, during annealing (Figure 3).

**Table 2:** LSPR wavelength (minima of transmission spectra) and corresponding FWHM.

	8 Pa				
	3 nm		6 nm		15 nm
	as deposited	500 °C, 2 h	as deposited	500 °C, 2 h	500 °C, 2 h

plasmon wavelength [nm]	553	569	637	560	575
FWHM [nm]	118	91	313	94	—

	12 Pa				

plasmon wavelength [nm]	579	549	615	562	569
FWHM [nm]	110	87	216	102	114

### Selection of the TiO_2_/Au surfaces for E2 detection

To test the TiO_2_/Au surfaces as SERS substrates, MBA was used as it is a well-known Raman reporter, showing two intense characteristic peaks at 1080 and 1590 cm^−1^ from aromatic ring vibrations [30]. The structure of MBA and the grafting process (thiol–gold interaction) guarantee that the molecule will preferentially attach to gold [37–38]. The purpose here was to select TiO_2_ growth and Au deposition parameters that would allow for the best enhancement possible over the largest wavelength range.

**Figure 5:**
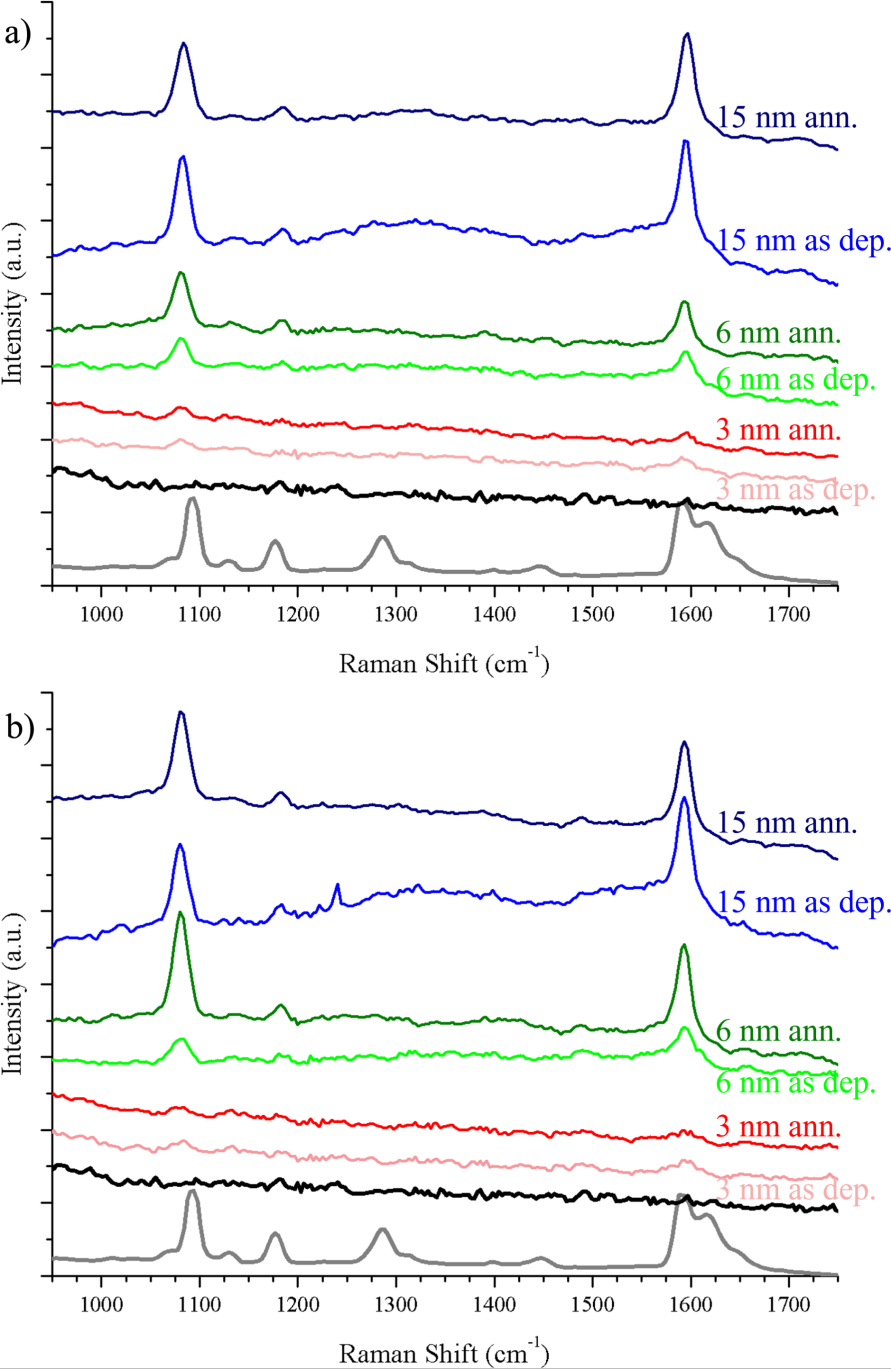
SERS measurements. The red, green and blue lines show SERS spectra of MBA deposited on, respectively, 3, 6, and 15 nm of Au deposited on TiO_2_ surfaces at 8 Pa (a) and 12 Pa (b). The corresponding brighter color means that there was no annealing of the TiO_2_. Grey line: Raman spectrum of MBA powder (the intensity has been scaled down to be comparable with the SERS spectra). Black line: spectrum of MBA deposited on a TiO_2_ surface without Au, yielding no SERS signal.

MBA was grafted on samples produced under the different growth conditions presented in the previous section. The detailed Raman spectra of MBA are displayed in Figure 5. MBA is barely detectable on the TiO_2_/Au 3 nm samples. The peaks start to be visible when the TiO_2_ surface is decorated with more than 6 nm of Au. A rule of thumb is that the highest enhancement is achieved when the wavelength of the LSPR is between the excitation wavelength (here 632 nm) and the Raman wavelength of the peak in consideration [5,39]. As shown in Figure 4 the plasmon resonances for all the samples were outside of this interval. The plasmonic properties of the NPs probably contribute to the enhancement of the Raman scattering. However, it also originates from the proximity of the NPs, which is higher for higher Au coverages (Table 1). The electric field between two nanoparticles is extraordinarily enhanced when the NPs are close to each other [7,40] and form so-called hot spots.

To compare the enhancement capacity of the TiO_2_/Au samples, the enhancement factor (EF) was calculated as follows [40]:

[1] $$EF=\frac{I_{\text{SERS}}}{I_{\text{Raman}}}\frac{N_{\text{Raman}}}{N_{\text{SERS}}}.$$

*I*_SERS_ and *I*_Raman_ are the intensities of the analyzed peak of MBA acquired in SERS configuration and from MBA powder in standard Raman configuration. *N*_Raman_ is the number of molecules within the excited laser volume, which can be calculated from the density and the molar mass of MBA (ρ_MBA_ = 1.5 g·cm^−3^, *M*_MBA_ = 154.19 g·mol^−1^), the laser spot area (*A*_spot_), and the penetration depth of the focused laser beam (which was assumed to be *h* =12 μm), as:

[2] $$M_{\text{Raman}}=A_{\text{spot}}h\frac{\rho_{\text{MBA}}}{M_{\text{MBA}}}N_{\text{A}},$$

where *N*_A_ is the Avogadro constant. *N*_SERS_ corresponds to the number of molecules adsorbed on the AuNP surface within the laser spot, which we assume to be a single monolayer of MBA fully covering the surface [38,41]. From the SERS and Raman spectra of Figure 5 the EF values were calculated (Table 3). The sample that gave the most homogeneous EF through over the investigated wavelength range was TiO_2_/Au 6 nm deposited at 12 Pa and annealed at 500 °C for 2 h. Hence, it was chosen for the subsequent examination of the of E2.

**Table 3:** Enhancement factor values for MBA, calculated for the peaks at 1080 and 1590 cm^−1^.

	8 Pa				
	Au 3 nm		Au 6 nm	Au 15 nm	
	as deposited	500 °C, 2 h	500 °C, 2 h	as deposited	500 °C, 2 h

1080 cm^−1^	1.9·10^5^	2.9·10^5^	5.2·10^5^	8.5·10^4^	2.5·10^5^
1590 cm^−1^	9.3·10^4^	8.2·10^4^	2.9·10^5^	5.0·10^4^	2.3·10^5^

	12 Pa				

1080 cm^−1^	1.5·10^5^	1.1·10^5^	3.7·10^5^	8.9·10^4^	3.4·10^5^
1590 cm^−1^	8.0·10^4^	3.9·10^4^	3.3·10^5^	6.0·10^4^	2.4·10^5^

	Si(100)				

1080 cm^−1^	—	—	2.7·10^4^	—	—
1590 cm^−1^	—	—	1.5·10^4^	—	—

**Figure 6:**
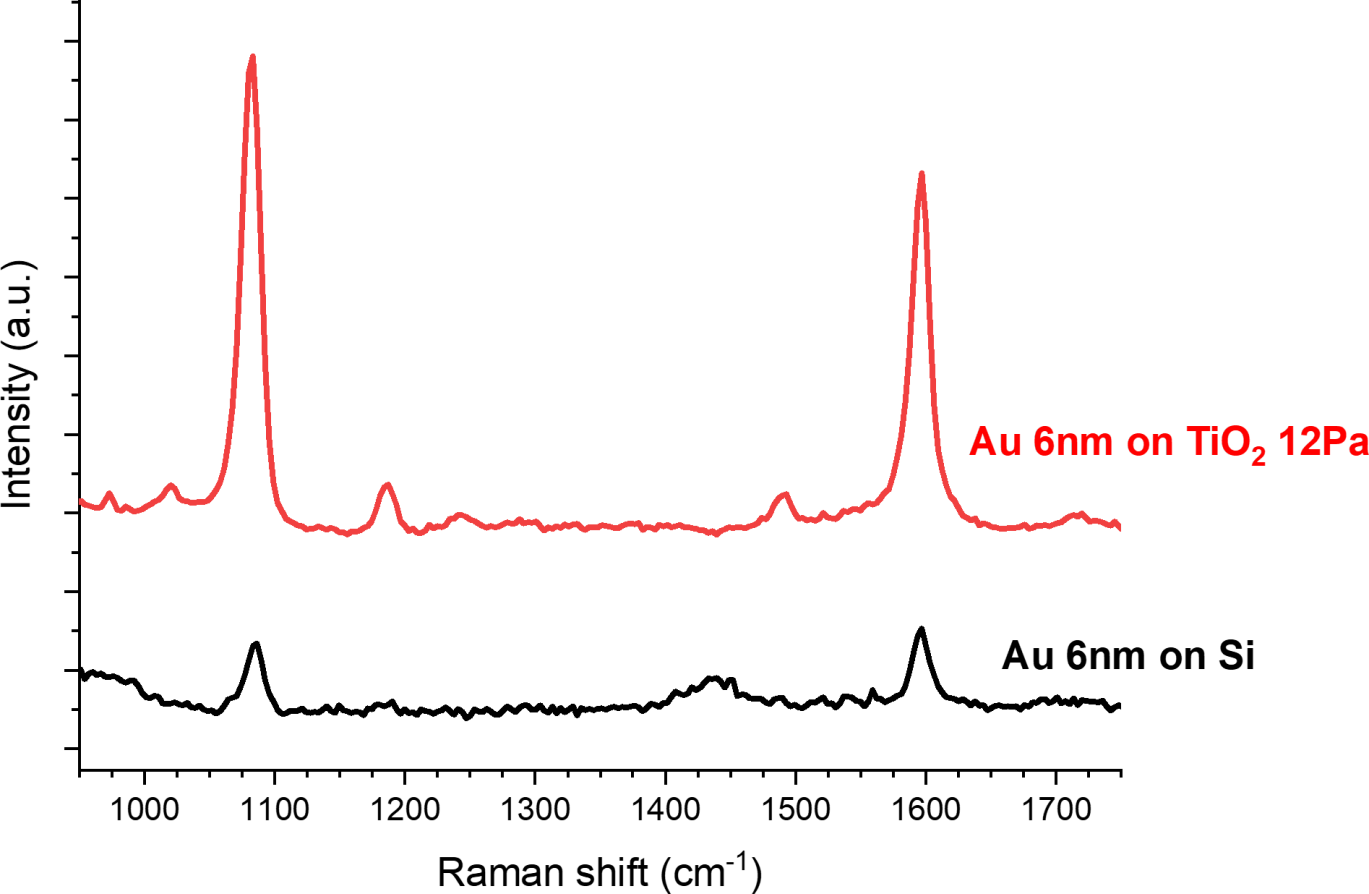
SERS enhancement measured for MBA of the same equivalent thickness of evaporated Au deposited on top of a bare Si(100) substrate (black line) and a nanostructured TiO_2_ film (red line).

Even though the SERS enhancement might be mainly attributed to an electromagnetic effect arising from the Au NPs, the presence of TiO_2_ could also be beneficial for different reasons. In addition to the abovementioned influence of the TiO_2_ surface on the size of the Au NPs (and consequently their plasmonic properties), a first hint can be found in the optical properties of semiconductor nanostructured materials. Their light-scattering, light-trapping and antireflection abilities, have already been reported to improve SERS enhancement [14–16]. In addition, the nanostructured morphology contributes to provide a larger available surface for Au NP growth, but also for analyte molecules to be adsorbed. The positive influence of the nanostructured TiO_2_ film was also confirmed by comparing SERS enhancements of the composite TiO_2_/Au 6 nm sensor with that of a bare Si(100) substrate on which the same equivalent thickness of Au (6 nm) was evaporated and annealed at 500 °C for 2 h. Figure 6 shows a clearly larger enhancement for both MBA peaks on the TiO_2_/Au sensor. The resulting EF is about one order of magnitude higher than that of the bare Si(100) substrate (Table 3). Therefore, the presence of the nanostructured TiO_2_ film benefits the overall SERS effect.

### Application to E2 detection

**Figure 7:**
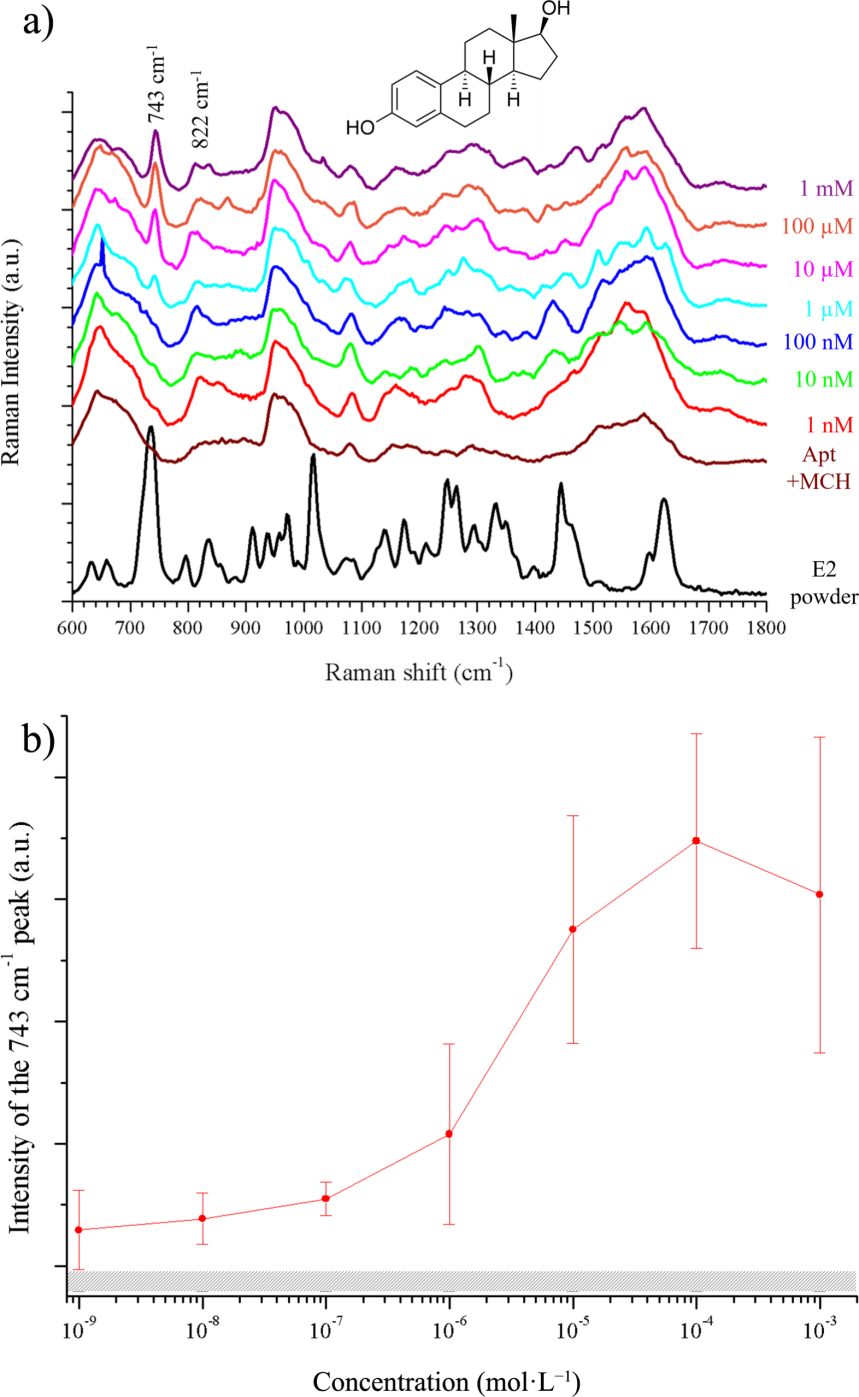
(a) Raman spectrum of E2 (black), SERS spectra of the empty sensor (Apt+MCH) and increasing concentrations of E2 on TiO_2_/Au 6 nm (annealed). The molecular structure of E2 is presented at the top. (b) Intensity of the peak at 743 cm^−1^ for increasing concentrations of E2. The hatched rectangle represents the intensity of the empty sensor and its uncertainty.

Figure 7a presents the Raman spectrum of E2 powder and the SERS spectra of E2 measured with the selected sensor. The signal of the empty sensor is designated as “Apt+MCH”. It mostly reflects the signal of the aptamer as MCH is known to have a very low Raman cross section and is thus not expected to yield a significant signal.

From the comparison between the spectra of the empty sensor (Apt+MCH) and of the hormone (E2 powder), it is possible to distinguish the zones where the E2 fingerprint can be found in the SERS spectra. These curves were acquired at concentrations ranging from 1 nM to 1 mM of E2. The fingerprint of E2 is clearly emerging from the aptamer spectra as the concentration increases. Shifts from the Raman spectra of the powder can be observed, which are due to the fact that the molecule is bound to the aptamer, affecting the vibration frequencies. For instance, the peak at 743 cm^−1^ corresponds to the peak at 730 cm^−1^ in the E2 Raman spectra, while the peak at 822 cm^−1^ corresponds to the peak at 830 cm^−1^. These two peaks are, respectively, attributed to bending of CCH and bending of CH [42]. They become clearer as the E2 concentration increases. Another feature can be seen in the region between 1200 and 1400 cm^−1^ corresponding to aromatic ring deformation modes, in-plane OH bending modes, and aliphatic/aromatic CH bending modes [43]. In order to study the evolution of the spectra with E2 concentration, we have plotted the total intensity of the peak at 743 cm^−1^ as a function of the E2 concentration (Figure 7b). The error bars reflect the homogenity of the sample surface. The signal increases with the concentration and reaches saturation at 100 µM. The hatched rectangle is the intensity of the same zone but for the empty sensor (without E2). Even at the lowest concentration (1 nM) the peak intensity is above this reference signal. The TiO_2_/Au 6 nm sensor was tested from 1 nM to 1 mM. The quantification is possible between 1 nM and 10 µM, i.e., the sensor has a dynamic range of at least four orders of magnitude.

## Conclusion

TiO_2_ nanoporous surfaces covered with Au NPs were tested as SERS surfaces for the detection of 17β-estradiol. Different conditions of Au deposition were considered as they lead to different shapes, sizes and distributions of the Au NPs. The TiO_2_/Au 6 nm deposited at 12 Pa and annealed for 2 h at 500 °C gives an enhancement factor (EF) of 3.7·10^5^ and 3.4·10^5^ at, respectively, 1080 and 1590 cm^−1^. These high EF values for two distant wavelengths has been exploited to test the detection of E2 in water. For the detection, the surfaces were functionalized with aptamers in order to guarantee a good specificity [17]. We thus have produced a sensor that is specific (with the use of aptamer), can detect low concentrations (1 nM, compatible with environmentally relevant concentration) and has a wide dynamic range (up to 100 µM). These results combined with the fact that the sensor is all solid makes the nanopourous TiO_2_/Au systems interesting for environmental detection applications.
